# Construction and application of an occupational health risk grading method based on risk matrix and dynamic weighting: a case study of footwear manufacturing enterprises

**DOI:** 10.3389/fpubh.2026.1895642

**Published:** 2026-09-03

**Authors:** Hongying Bian, Xin Wang, Yiwen Dong, Ning Kang, Lin Zhang

**Affiliations:** National Institute of Occupational Health and Poison Control, Chinese Center for Disease Control and Prevention & Chinese Academy of Preventive Medicine, Beijing, China

**Keywords:** dynamic weighting, footwear manufacturing enterprise, occupational health, risk assessment, risk matrix

## Abstract

**Background:**

Occupational hazard factors in the footwear manufacturing industry are complex and encompass benzene series compounds, ketones, esters, dust, noise, and other agents. Existing mainstream occupational health risk assessment methods—including the Singapore semi-quantitative risk assessment method (Ministry of Manpower method, hereafter referred to as the MOM method), the exposure ratio method specified in GBZ/T 298-2017 (hereafter referred to as the exposure ratio method), the United States Environmental Protection Agency (EPA) inhalation risk assessment method (hereafter referred to as the EPA method), and the International Council on Mining and Metals (ICMM) occupational health risk assessment model (hereafter referred to as the ICMM method)—each have specific applicable scenarios and limitations. No single method can achieve precise risk grading and dynamic management.

**Objective:**

To construct a novel occupational health risk grading method that integrates the strengths of four mainstream methods, based on a risk matrix and dynamic weighting, and to validate its applicability using footwear manufacturing enterprises as a case study.

**Methods:**

Four footwear manufacturing enterprises (A, B, C, D) were selected, and on-site measurements of chemical toxicants, dust, and noise in the workplace were conducted. Risk assessments were performed using the MOM method, the exposure ratio method, the EPA method, and the ICMM method. A risk ratio normalization approach was applied to align the risk levels from all methods into a unified five-level system. On this basis, a risk matrix was constructed, and a combined weighting approach integrating the entropy weight method and the analytic hierarchy process (AHP) was employed to determine the dynamic weights of each method under different hazard scenarios. A comprehensive risk index (CRI) was constructed and graded. Consistency among methods was analyzed using Spearman’s rank correlation, weighted Kappa coefficient, Fleiss’ Kappa coefficient, and Wilcoxon signed-rank test.

**Results:**

Maximum benzene concentrations reached 3.41 mg/m^3^ (Enterprise B) and 5.7 mg/m^3^ (Enterprise C, exceeding the limit); methyl ethyl ketone peaked at 175.0 mg/m^3^ (Enterprise D). The conventional methods showed moderate agreement (Fleiss’ Kappa = 0.455). CRI demonstrated substantial agreement with each method (*κ*_*w* = 0.625–0.796) and significantly moderated ICMM overestimation (*Z* = −2.873108, *p* = 0.004). CRI classified Enterprise C gluing as Level V (CRI = 1.00) and Enterprise D benzene as Level II (CRI = 0.28). Dust yielded a maximum CRI of 0.42 (medium risk). Noise CRI reached 0.60 (medium risk, Level III). Sensitivity analysis confirmed robustness of *α*, *β*, *γ* coefficients.

**Conclusion:**

The constructed grading and classification method can more objectively and comprehensively reflect the actual occupational health risks in footwear manufacturing enterprises, providing a scientific basis for formulating differentiated and precise management strategies.

## 1 Introduction

### 1.1 Research background and significance

Conducting graded and classified assessments for enterprises with different characteristics can improve the precision and efficiency of occupational health management by employers and safeguard the health rights of workers (1). The core principle of grading and classification is to evaluate and rank the comprehensive health risks faced by occupational populations through rational and effective occupational health risk assessment methods, distinguishing primary risks from secondary risks. By integrating the occupational health management status of employers, reasonable classification can be achieved, facilitating targeted supervision by management authorities (2). The key to establishing an occupational health grading and classification method is selecting appropriate and effective risk assessment methods to comprehensively evaluate the occupational health risks of different positions, as the classification assessment of enterprise management status is relatively simple and standardized based on national regulations or industry standards.

Currently, widely used international occupational health risk assessment methods include the MOM method (3) for chemical toxicants, “Technical Guide for Occupational Health Risk Assessment of Chemical Hazardous Factors in the Workplace” (GBZ/T 298) (4), the United States Environmental Protection Agency (EPA) inhalation risk assessment guidelines (5), and the International Council on Mining and Metals (ICMM) occupational health risk assessment guidelines (6). These methods each have certain advantages and disadvantages. Numerous scholars worldwide have validated these existing methods. The MOM method and the exposure ratio method are relatively simple and practical (7); the EPA method is relatively accurate for quantitative assessment of chemical toxicants (8); the ICMM method has certain advantages in assessing chemical toxicants, dust, and physical factors (9). The aforementioned four methods each have their applicable scenarios and limitations, and a single method cannot achieve precise grading and dynamic management.

The footwear manufacturing industry is an important part of China’s labor-intensive manufacturing sector. Its production process involves multiple operations such as cutting, leather forming, upper making, sole making, painting, and finished product packaging. Enterprise scales vary significantly, ranging from large enterprises with thousands of employees to numerous small enterprises with fewer than 100 employees. Small enterprises often have only one comprehensive production workshop and a chemical warehouse, while large enterprises are divided into independent units such as main production workshops, chemical warehouses, and wastewater treatment stations based on functions. Large quantities of adhesives, treatment agents, inks, and organic solvents are used during production, resulting in the ubiquitous presence of benzene series, ketones, esters, dust, and noise in the workplace air. Cases of benzene poisoning have been reported (10, 11).

This study uses the footwear manufacturing industry as a case, applies four internationally commonly used risk assessment methods to assess the occupational health risks of corresponding positions, and compares their advantages and disadvantages. On this basis, a risk matrix is constructed, and a combined weighting method integrating the entropy weight method (12, 13) and the analytic hierarchy process (AHP) (14, 15) is used to determine the dynamic weights of each method under different hazard scenarios. A comprehensive risk index (CRI) is established and graded, forming an occupational health graded and classified management method. By evaluating the occupational health risks of chemical toxicants, dust, and noise exposure at key positions in footwear enterprises of different scales, this study aims to help managers understand the current status of occupational health hazard risks, provide a reference for supervision and management, protect workers’ health, and reduce the occurrence of occupational diseases.

### 1.2 Research status and problem statement

In recent years, scholars both domestically and internationally have conducted extensive research on occupational hazard risk assessment in the footwear manufacturing industry. Abouee-Mehrizi et al. (16) assessed the health risks of exposure to benzene, toluene, ethylbenzene, and xylene in footwear workplaces and found that combined exposure to multiple organic solvents increased the carcinogenic and non-carcinogenic risks to workers. Azari et al. (17) evaluated the exposure of shoemakers to benzene and toluene compounds in small footwear workshops in East Tehran, revealing weaknesses in occupational health management in small and medium-sized footwear enterprises. Domestically, Fu et al. (18) used the MOM method to assess the risk levels of key hazard factors in 195 enterprises in Zhongshan city. Peng et al. (19) compared the application of multiple occupational health risk assessment methods in four footwear enterprises in Guangzhou and found that the ICMM method differed significantly from other methods. Comparative studies of multiple risk assessment methods are increasingly enriched. Ye et al. (20) applied three semi-quantitative methods from GBZ/T 298 in the automobile manufacturing industry and found significant differences in consistency among different methods (weighted Kappa = 0.118–0.136). Shi et al. (21) compared four methods in dust-exposed positions of pipeline anti-corrosion enterprises and found that the ICMM method yielded significantly higher assessment results than other methods, with a Fleiss’ Kappa value of −0.274. Li et al. (22) conducted occupational health risk assessment and risk monetization based on xylene exposure, showing that the EPA and the MOM method were relatively reasonable, while the ICMM model tended to overestimate risks. The above studies reveal a core problem: the four methods have their respective strengths, but there is a lack of a unified framework that can dynamically integrate assessment results based on specific hazard characteristics and exposure scenarios.

### 1.3 Research objectives and innovations

This study aims to construct a novel occupational health graded and classified management method based on a “risk matrix” and “dynamic weighting,” and to validate its applicability using four footwear manufacturing enterprises of different scales as cases. The innovations include: (1) Method integration: for the first time, organically integrating four mainstream methods into a comprehensive assessment framework; (2) Dynamic weighting: introducing a combined weighting strategy that integrates objective weighting (entropy weight method) and subjective weighting (AHP), effectively solving the decision-making problem when results from multiple methods conflict; (3) Empirical validation: systematic validation based on actual monitoring data (covering diverse data scenarios with a large proportion of values below the detection limit and exceeding the limit), and consistency evaluation using multiple statistical methods.

## 2 Materials and methods

### 2.1 Research subjects

Four footwear manufacturing enterprises (coded A, B, C, D) were selected as research subjects, covering different scales, process flows, and product types. The production processes of the four enterprises were basically the same: cutting → leather forming → upper making → sole making → painting → finished product → packaging.

#### 2.1.1 Enterprise profiles

*Enterprise A*: Small enterprise with 23 employees. Only one comprehensive production workshop and a chemical warehouse. Mainly produces leather shoes, with manual and semi-automatic operations.

*Enterprise B*: Small enterprise with 60 employees. Only one comprehensive production workshop and a chemical warehouse. Mainly produces sports shoes, with functional areas for leather shoe production, shoe strip production, shoe box production, screen printing, and auxiliary production.

*Enterprise C*: Small enterprise with 97 employees. Only one comprehensive production workshop (including upper making workshop and sole workshop) and a chemical warehouse. Mainly produces leather shoes.

*Enterprise D*: Large enterprise with approximately 4,000 employees. Functionally divided into five main production workshops (A, B, C, D, E), a chemical warehouse, and a wastewater treatment station. Production processes include cutting, molding, painting, printing, adhesive mixing, rubber mixing, and injection molding.

Through on-site occupational health investigations, the main occupational hazard factors identified in the four enterprises included: chemical toxicants—benzene, toluene, xylene, ethylbenzene, ethyl acetate, butyl acetate, acetone, methyl ethyl ketone, n-hexane, formaldehyde, vinyl chloride, TDI, methanol, cyclohexanone, carbon tetrachloride, etc.; dust—leather dust, other dust; physical factors—noise.

### 2.2 On-site monitoring and data sources

#### 2.2.1 Sampling and analysis methods

On-site sampling was conducted in accordance with GBZ 159-2004 “Specifications of Air Sampling for Hazardous Substances Monitoring in the Workplace,” using a combination of individual sampling and area sampling. Sampling followed GBZ 159-2004 (23). Analysis was conducted according to GBZ/T 192.1-2007 (24), GBZ/T 300.99-2017 (25), GBZ/T 300.66-2017 (26), GBZ/T 300.122-2017 (27), GBZ/T 160.63-2004 (28), and GBZ/T 300.60-2017 (29). Monitoring was conducted for leather dust, other dust, welding fume, formaldehyde, benzene, toluene, xylene, ethyl acetate, butyl acetate, acetone, methyl ethyl ketone, cyclohexanone, methanol, propanol, cyclohexane, n-hexane, dichloromethane, carbon tetrachloride, hydrochloric acid, acrylic acid, styrene, toluene diisocyanate (TDI), sulfur dioxide, hydrogen sulfide, manganese and its compounds, nitrogen oxides, carbon monoxide, ammonia, ozone, chlorine dioxide, etc., in the workplace air. Noise intensity in the workplace was measured according to GBZ/T 189.8-2007 (30).

#### 2.2.2 Descriptive statistics of monitoring data

The monitoring results of the main occupational hazard factors in the four enterprises were processed and described statistically. Because the monitoring results of the same hazard factor at different positions did not conform to a normal distribution (Shapiro–Wilk test, *p* < 0.05 for all variables), they are presented as median *M* (P25, P75); for hazard factors with fewer than three monitoring results, specific values are listed directly.

#### 2.2.3 Data preprocessing

For data below the limit of detection (LOD), 1/2 LOD was used as a substitute for statistical analysis. For multiple monitoring results at the same position, the time-weighted average concentration (TWA) or the average of TWA values was used for risk assessment. Noise data were uniformly converted to 8 h equivalent continuous A-weighted sound pressure level (LEX,8h) and compared with the occupational exposure limit of 85 dB (A).

### 2.3 Four benchmark risk assessment methods

#### 2.3.1 The MOM method

The MOM method calculates the risk value based on the hazard rating (HR, Levels 1–5) and exposure rating (ER, Levels 1–5) of chemical substances: Risk = HR × ER. HR is determined based on toxicological data, carcinogenicity classification, acute toxicity level, etc.; ER is determined by comprehensively considering the ratio of measured concentration to OEL, exposure frequency, exposure duration, and engineering control measures. The risk value is divided into five levels: Risk ≤2 is Level I (negligible risk), 3–6 is Level II (low risk), 7–12 is Level III (medium risk), 13–20 is Level IV (high risk), and 21–25 is Level V (extremely high risk).

#### 2.3.2 The exposure ratio method

The exposure ratio method, based on GBZ/T 298-2017, uses the ratio of exposure concentration (*E*) to the occupational exposure limit (OEL) (*E*/OEL) as the core indicator for risk level determination. The *E*/OEL ratio is divided into five levels: *E*/OEL < 0.1 is Level 1 (negligible risk); 0.1 ≤ *E*/OEL < 0.5 is Level 2 (low risk); 0.5 ≤ *E*/OEL < 1.0 is Level 3 (medium risk); 1.0 ≤ *E*/OEL < 2.0 is Level 4 (high risk); *E*/OEL ≥ 2.0 is Level 5 (extremely high risk).

#### 2.3.3 The EPA method

The EPA inhalation risk assessment model is applicable to carcinogens with known inhalation unit risk (IUR) values (e.g., benzene). The carcinogenic risk (CR) is calculated as: CR = EC × IUR. To maintain consistency with the other five-level methods, this study refines the risk determination criteria into five levels: CR ≤ 1 × 10^−6^ is Level 1 (negligible risk); 1 × 10^−6^ < CR ≤ 1 × 10^−5^ is Level 2 (low risk); 1 × 10^−5^ < CR ≤ 1 × 10^−4^ is Level 3 (medium risk); 1 × 10^−4^ < CR ≤ 1 × 10^−3^ is Level 4 (high risk); CR > 1 × 10^−3^ is Level 5 (extremely high risk).

#### 2.3.4 The ICMM method

The ICMM occupational health risk assessment model includes a qualitative matrix method and a quantitative method. The qualitative matrix method constructs a risk assessment matrix based on the severity of health consequences and the probability of exposure. The quantitative method further considers exposure time, calculating risk value = consequence × exposure probability × exposure time. Risk levels range from Level 1 (tolerable risk) to Level 5 (intolerable risk).

### 2.4 Risk level coding rules

All methods ultimately adopt a unified five-level risk system (Level I is the lowest, Level V is the highest). The level coding rules are as follows (Table 1).

**Table 1:** Coding rules for the five-level risk system.

Level name	Code	Score assignment
Negligible/no hazard	Level I	1
Low risk/general hazard	Level II	2
Medium risk	Level III	3
High risk	Level IV	4
Extremely high risk/intolerable	Level V	5

### 2.5 Novel graded and classified management method based on risk matrix and dynamic weighting

#### 2.5.1 Risk matrix construction

A 5 × 5 risk matrix was constructed as the benchmark framework for comprehensive assessment: *X*-axis (Severity of Health Consequences): Comprehensively considers the toxicological characteristics of chemical substances, including carcinogenicity classification, acute toxicity level, sensitization, reproductive and developmental toxicity, target organ toxicity, etc., divided into levels 1–5.

*Y*-axis (Probability of Exposure): Comprehensively considers the ratio of on-site measured concentration to OEL, exposure frequency and duration, effectiveness of engineering control measures, use of personal protective equipment, etc., divided into levels 1–5.

The matrix grid is divided into five risk levels (Level I negligible risk to Level V extremely high risk).

#### 2.5.2 Dynamic weighting model

##### 2.5.2.1 Weight factor system

A three-dimensional correction framework incorporating hazard characteristic (*α*), data reliability (*β*), and scenario applicability (*γ*) factors was established to dynamically optimize method weights.

*α-Hazard characteristic factor*: Predefined assignment principles were formulated prior to data analysis for carcinogenic and non-carcinogenic assessment scenarios separately. For carcinogen evaluation: The target enhancement level of the EPA method was preset at *α* = 1.5, based on established toxicological consensus that the EPA inhalation unit risk model serves as the standard approach for quantitative carcinogen evaluation. For the remaining three methods (MOM, exposure ratio method, ICMM method), their feasible range of α was predefined as 1.0–1.5. A clear prior rule was specified: the relative magnitude of *α* should match the comprehensive ranking derived from subsequently calculated entropy weights and AHP subjective weights. Methods with higher comprehensive weight rankings would be assigned larger enhancement coefficients, while methods with weaker discrimination capacity and suitability adopted the baseline value of 1.0. After entropy weights and AHP weights were obtained, the final α values were determined strictly following the above principles.

For non-carcinogenic threshold toxicants: The exposure ratio method and MOM method take the ratio of measured concentration to occupational exposure limit as core evaluation indicators and were prioritized for enhancement with *α* = 1.5. The EPA inhalation unit risk model lacks mature evaluation logic applicable to threshold pollutants. Although the ICMM method exhibits relatively wide universality, its risk discrimination performance is insufficient for threshold pollutants. Therefore, both the EPA method and ICMM method were set to the baseline value of 1.0 under non-carcinogenic scenarios.

*β-data reliability factor*: A baseline value of 1.0 was adopted for all methods under complete and reliable monitoring data. When more than 50% of EPA monitoring samples fell below the detection limit, *β* would be reduced to mitigate uncertainty of quantitative carcinogen risk estimation induced by censored data. No upward adjustment of *β* was designed in the present study.

*γ-method applicability factor*: *γ* coefficients were allocated according to temporal characteristics of exposure based on the inherent response characteristics of each assessment method.

For short-term high-concentration peak exposure: The exposure ratio method directly responds to instantaneous concentration surges and shows the strongest sensitivity to acute peaks, hence *γ* = 1.5; the EPA method can reflect short-term exposure risk but is less sensitive to transient fluctuations, therefore mild enhancement was adopted with *γ* = 1.2.

For long-term low-concentration exposure: The EPA and ICMM methods are suitable for evaluating cumulative and chronic adverse effects, so both were enhanced with *γ* = 1.4. The MOM method cannot reflect the influence of exposure duration and was set to maintain the baseline level *γ* = 1.0 across all exposure scenarios.

After confirming all three-dimensional correction coefficients, normalized dynamic weights for CRI calculation were calculated as follows:


$$W_{\mathrm{dyn},j}=\frac{W_{\mathrm{com},j}\times \alpha_{j}\times \beta_{j}\times \gamma_{j}}{\sum (W_{\mathrm{com},j}\times \alpha_{j}\times \beta_{j}\times \gamma_{j})}$$

Sensitivity analysis was implemented to verify model robustness. Each correction coefficient was varied within ±20% of the finalized values. The results confirmed that categorical risk levels of all representative positions remained unchanged, indicating reliable stability of the evaluation framework.

##### 2.5.2.2 Combined weighting approach

Objective weighting (entropy weight method): Based on the assessment result matrix of the four methods for each position/hazard factor in the four enterprises, the objective weight of each method is determined using the information entropy principle (see Statistical Analysis Methods Section for detailed formulas).

Subjective weighting (AHP method): 5–7 experts in the field of occupational health are invited to determine the subjective weight of each method using the 1–9 scale method. A judgment matrix was constructed based on the pairwise comparisons, and the AHP weights were derived by calculating the eigenvector of the matrix. Specifically, the weight vector *w* = (*w*_1_, *w*_2_, …, *w_m_*)^T^ was obtained from the equation *A_w_* = *λ*_max_*w*, where *A* = (*a_ij_*)_*m*×*m*_ is the judgment matrix, *λ*_max_ is the maximum eigenvalue, and *m* is the number of methods. The consistency index (CI) was calculated as CI = (*λ*_max_ − *m*)/(*m* − 1), and the consistency ratio (CR) was determined as CR = CI/RI, where RI is the random index.

Combined weighting: *w_j_*_combined = 0.5 × *w_j_*_entropy + 0.5 × *w_j_*_AHP, further corrected by multiplying by the three-dimensional factors.

##### 2.5.2.3 Comprehensive risk index (CRI) calculation

CRI = *w*₁ × *S*₁ + *w*₂ × *S*₂ + *w*₃ × *S*₃ + *w*₄ × *S*₄, where *S*₁–*S*₄ are the normalized risk scores (0-1) of the four methods, and *w*₁–*w*₄ are the dynamic weights (summing to 1).

*Weight normalization for noise-exposed positions*: For noise-exposed positions, because only the ICMM method yielded valid assessments among the four benchmark methods, the dynamic weighting model normalized the ICMM weight to 1.0. Accordingly, the CRI for noise positions equals the ICMM normalized score: CRInoise = *S*_ICMM_. While multi-method cross-validation is not feasible for noise, the dynamic method still enables comparative analysis through: (i) intra-enterprise inter-position comparisons, (ii) inter-enterprise comparisons, and (iii) reference threshold-based gradient comparisons.

##### 2.5.2.4 Grading standards and corresponding management strategies

Based on the CRI value and risk matrix results, occupational health risks are divided into five levels, and corresponding graded management strategies and a dynamic adjustment mechanism are formulated: The comprehensive risk classification results should be subject to periodic assessment and dynamic adjustment. When the occupational disease hazard risk level of an enterprise changes, the comprehensive risk category should be adjusted accordingly (Table 2).

**Table 2:** Risk grading and corresponding management strategies of the new method.

Risk level	CRI range	Risk description	Management measures
Level I	0.0–0.2	Negligible risk	Maintain routine monitoring, retest annually; maintain existing control measures
Level II	0.2–0.4	Low risk	Strengthen maintenance of protective facilities, monitor every 6 months; improve personal protection training
Level III	0.4–0.6	Medium risk	Optimize local protective facilities, monitor quarterly; improve engineering control measures
Level IV	0.6–0.8	High risk	Comprehensively renovate protective facilities, rectify within a limited time
Level V	0.8–1.0	Extremely high risk	Immediately suspend production for rectification or position adjustment; mandatory personal protection; emergency management

CRI values at the cut‑off points are assigned to the lower risk level.

### 2.6 Statistical analysis methods

SPSS 26.0 and R (Version 4.4.3) were used for statistical analysis. Normality was tested using the Shapiro–Wilk test. Consistency among methods was analyzed using the following methods:

1. Spearman’s rank correlation coefficient: evaluates the correlation between the assessment results of two methods (31). The strength of correlation is classified based on |*r_s_*|: 0.8–1.0 very strong, 0.6–0.8 strong, 0.4–0.6 moderate, 0.2–0.4 weak, <0.2 very weak. Significance level *α* = 0.05.
2. Weighted Kappa coefficient: evaluates the degree of agreement between the assessment results of two methods, considering the severity of disagreement between levels. Based on the standards of Landis and Koch (32) and Cohen (33): *K* < 0 poor, 0.0–0.20 slight, 0.21–0.40 fair, 0.41–0.60 moderate, 0.61–0.80 substantial, 0.81–1.00 almost perfect. The formula for weighted Kappa coefficient incorporates observed agreement (*P*0) and expected agreement (*Pe*). For small samples, bootstrap resampling (34, 35) was adopted to mitigate small-sample bias. Specifically, we performed 1,000 times of with-replacement random sampling from the original subset, computed weighted Kappa for every bootstrap sample, and used the 2.5th and 97.5th percentile values of the bootstrap Kappa distribution to determine the final 95% confidence interval.
3. Fleiss’ Kappa coefficient: evaluates the overall agreement among multiple methods.
4. Wilcoxon signed-rank test: compares the difference in the distribution of assessment levels between the new method and each conventional method.
5. Entropy weight method formulas:
  ① Original data matrix: $X={\left(x_{\mathrm{ij}}\right)}_{n\times 4}$
  ② Data normalization: $p_{\mathrm{ij}}=\frac{x_{\mathrm{ij}}}{\sum_{i=1}^{n}x_{\mathrm{ij}}}$
  ③ Information entropy: $e_{j}=-\frac{1}{\ln(n)}\sum_{i=1}^{n}p_{\mathrm{ij}}\ln(p_{\mathrm{ij}})$
  ④ Entropy weight: $w_{j}=\frac{1-e_{j}}{\sum_{j=1}^{4}(1-e_{j})}$
  where: *n* is the number of representative positions assessed in this study. Methods with smaller (*e_j_*) provide greater information discrimination and thus receive higher weights.

## 3 Results

### 3.1 Main occupational hazard factor monitoring results of the four enterprises

The monitoring results of the four enterprises showed: In Enterprise A, the maximum toluene concentration was 44.7 mg/m^3^, approaching the limit of 50 mg/m^3^; in Enterprise B, the maximum benzene concentration was 3.41 mg/m^3^, exceeding the limit; in Enterprise C, the benzene concentration at the gluing position was 5.7 mg/m^3^, exceeding the limit; in Enterprise D, benzene was not detected in all samples, but the peak concentration of methyl ethyl ketone was 175 mg/m^3^ (*E*/OEL = 0.58), exceeding the action level (Table 3).

**Table 3:** Summary of main occupational hazard factor monitoring results of the four enterprises (mg/m^3^, noise in dB (A)).

Enterprise	Hazard factor	No. of monitoring points (*n*)	*M* (P25, P75) or specific values (mg/m^3^)/dB (A)	Maximum (mg/m^3^)/dB (A)	PC‑TWA (mg/m^3^)/ LEX,8h(dB (A))	No. of points exceeding limit
A	Dust (leather, other)	2	0.13 (0.10, 0.15)	0.15	8	0
A	Benzene	4	0.75 (0.25, 0.95)	1.2	3.0	0
A	Toluene	4	16.85 (5.20, 37.15)	44.7	50	0
A	Xylene	4	0.30 (0.2, 0.45)	0.9	50	0
A	Ethyl acetate	4	3.40 (1.4, 5.85)	6.9	200	0
A	Butyl acetate	4	0.15 (0.1, 0.3)	0.7	200	0
A	n-Hexane	2	<0.3, <0.3	<0.3	100	0
A	Acetone	4	2.75 (1.3, 5.35)	7.9	300	0
A	Methyl ethyl ketone	4	2.00 (1.0, 3.75)	5.8	300	0
A	Formaldehyde	2	0.23, 0.16	0.23	0.5	0
A	Noise	5	76.1 (74.0, 78.8)	79.4	85	0
B	Dust (other)	1	0.16	0.16	8	0
B	Benzene	7	0.45 (0.24, 0.58)	3.41	3.0	1*
B	Toluene	7	1.17 (0.56, 1.57)	2.13	50	0
B	Xylene	7	<0.04 (all)	<0.04	50	0
B	Ethyl acetate	7	<0.15 (all)	<0.15	200	0
B	Butyl acetate	7	<0.08 (all)	<0.08	200	0
B	n-Hexane	7	<0.06 (all)	<0.06	100	0
B	Acetone	7	0.23 (0.14, 0.31)	0.32	300	0
B	Methyl ethyl ketone	7	0.27 (0.15, 0.36)	0.63	300	0
B	Formaldehyde	5	<0.11 (all)	<0.11	0.5	0
B	Vinyl chloride	1	<0.07	<0.07	10	0
B	Carbon tetrachloride	1	5.68	5.68	15	0
B	Hydrochloric acid	1	0.95	0.95	7.5	0
B	Noise	19	75.0 (73.9, 76.3)	77.6	85	0
C	Dust (leather)	3	0.5 (0.4, 0.6)	0.6	8	0
C	Benzene	5	0.9 (0.9, 1.4)	5.7	3.0	1*
C	Toluene	5	0.9 (0.5, 3.4)	11.5	50	0
C	Xylene	5	<0.4 (<0.4, 10.8)	10.8	50	1
C	Ethylbenzene	5	<0.2 (<0.2, 15.4)	15.4	100	1
C	Ethyl acetate	5	0.9 (0.7, 1.9)	2.7	200	0
C	Butyl acetate	5	0.6 (0.6, 4.3)	13.6	200	0
C	Noise	19	72.4 (70.4, 77.8)	83.8	85	0
D	Dust (leather/other)	6	0.6 (0.4, 1.1)	1.2	8	0
D	Benzene	15	<0.04 (all)	<0.04	3.0*	0
D	Toluene	15	0.4 (0.11, 0.5)	8.0	50	0
D	Xylene	15	<0.2 (all)	<0.2	50	0
D	Ethyl acetate	15	1.52 (0.72, 17.4)	82.5	200	0
D	Butyl acetate	15	<0.02 (<0.02, 2.86)	2.86	200	0
D	Acetone	15	1.9 (0.41, 4.31)	10.8	300	0
D	Methyl ethyl ketone	15	1.38 (0.76, 4.03)	175.0	300	0
D	TDI	3	<0.0002 (all)	<0.0002	0.1	0
D	Methanol	4	2.73 (0.17, 24.4)	24.4	25	0
D	Cyclohexanone	4	0.98 (0.18, 2.37)	2.37	50	0
D	Noise	31	79.2 (76.4, 82.2)	84.8	85	0

*The PC-TWA for benzene is 3.0 mg/m^3^ (GBZ 2.1-2019 amendment no. 1, implemented in 2021). The overall median benzene concentration in Enterprise B was 0.45 mg/m^3^, but the maximum was 3.41 mg/m^3^; the maximum in Enterprise C was 5.7 mg/m^3^.

#### 3.1.1 Assessment results of the four conventional methods

In the assessment of representative positions in the four enterprises, both the MOM method and the exposure ratio method rated benzene in Enterprise C as Level V, and toluene in Enterprise A as Level III. The ICMM method rated dust in Enterprise A as Level V, while the MOM method and exposure ratio method rated it as Level I. For xylene in Enterprise C, the MOM method and exposure ratio method rated it as Level II, while the ICMM method rated it as Level V. Regarding noise, the noise at the rubber mixing noise in Enterprise D (84.8 dB (A)) was rated as Level III by the ICMM method. The EPA method was only applicable to benzene; the benzene exposure in Enterprise C was rated as Level V, consistent with the ICMM method (Table 4).

**Table 4:** Comparison of risk level assessment of representative positions by four methods.

Enterprise	Position	Hazard factor	Measured value	E/OEL	MOM method	Exposure ratio method	EPA method	ICMM method
A	Molding/Spray gluing	Toluene	44.7 mg/m^3^	0.89	Level III	Level III	—	Level V
A	Molding/Spray gluing	Benzene	1.2 mg/m^3^	0.60	Level III	Level III	Level III	Level V
A	Cutting	Dust	0.15 mg/m^3^	0.019	Level I	Level I	—	Level V
A	Stitching	Noise	79.4 dB (A)	0.93	—	—	—	Level II
B	Printing	Benzene	3.41 mg/m^3^	1.71	Level IV	Level IV	Level IV	Level IV
B	Spray gluing	Benzene	2.28 mg/m^3^	1.14	Level IV	Level IV	Level IV	Level IV
B	Injection molding	Carbon tetrachloride	5.68 mg/m^3^	0.38	Level II	Level II	—	Level III
B	Cutting	Noise	77.6 dB (A)	0.91	—	—	—	Level III
C	Gluing	Benzene	5.7 mg/m^3^	2.85	Level V	Level V	Level V	Level V
C	Gluing	Xylene	10.8 mg/m^3^	0.22	Level II	Level II	—	Level V
C	Gluing	Ethylbenzene	15.4 mg/m^3^	0.15	Level II	Level II	—	Level V
C	Spray polishing	Butyl acetate	13.6 mg/m^3^	0.068	Level I	Level I	—	Level III
C	Cutting	Dust	0.60 mg/m^3^	0.075	Level I	Level I		Level IV
C	Machine sewing	Noise	83.8 dB (A)	0.99	—	—	—	Level II
D	Painting	Benzene	<0.04 mg/m^3^	<0.02	Level I	Level I	Level I	Level III
D	Printing	Methyl ethyl ketone	175 mg/m^3^	0.58	Level III	Level III	—	Level V
D	Printing	Ethyl acetate	82.5 mg/m^3^	0.41	Level II	Level II	—	Level IV
D	Cutting	Dust	1.2 mg/m^3^	0.15	Level I	Level II		Level IV
D	Rubber mixing	Noise	84.8 dB (A)	1.00	—	—	—	Level III
D	Cutting	Noise	84.6 dB (A)	1.00	—	—	—	Level III

### 3.2 Dynamic weight determination results

#### 3.2.1 Objective weights via entropy weight method

The normalized levels of the four methods (Level I = 1, II = 2, III = 3, IV = 4, V = 5) for 23 representative positions were used to construct the original data matrix (23 × 4), and the entropy weight method was applied to calculate the objective weights of each method. Results showed that the exposure ratio method had the smallest information entropy (0.723), the largest redundancy (0.277), and an entropy weight of 0.305, indicating the strongest ability to distinguish the risk levels of different positions; the entropy weights of the ICMM method, MOM method, and EPA method were 0.269, 0.233, and 0.193, respectively (Table 5).

**Table 5:** Calculation process and results of the entropy weight method.

Method	Information entropy *eⱼ*	Redundancy *dⱼ*	Entropy weight (*W*_entropy)
Exposure ratio method	0.723	0.277	0.305
ICMM method	0.756	0.244	0.269
MOM method	0.789	0.211	0.233
EPA method	0.825	0.175	0.193

#### 3.2.2 Subjective weights via AHP

For the carcinogen assessment scenario (e.g., benzene, formaldehyde), five occupational health experts (from CDC, occupational health testing agencies, universities, and enterprises) were invited to conduct pairwise comparisons of the relative importance of the four methods, constructing a judgment matrix. The calculated weight was highest for the EPA method (0.490), followed by the exposure ratio method (0.271), MOM method (0.146), and ICMM method (0.093). The consistency ratio (CR) of the judgment matrix was 0.016 < 0.10, passing the consistency test (Table 6).

**Table 6:** AHP judgment matrix and weights for carcinogen assessment scenario.

Method	MOM method	Exposure ratio method	EPA method	ICMM method	Weight (*W*_AHP)
EPA method	4	2	1	4	0.490
Exposure ratio method	2	1	1/2	3	0.271
MOM method	1	1/2	1/4	2	0.146
ICMM method	1/2	1/3	1/4	1	0.093

*λ*max = 4.043, CI = 0.014, RI = 0.90, CR = 0.016.

#### 3.2.3 Combined weights and three-dimensional factor correction

The linear weighted combination method (*λ* = 0.5) was used to merge the entropy weight and AHP weight, obtaining the combined weight *W*_combined = 0.5 × *W*_entropy + 0.5 × *W*_AHP. On this basis, the hazard characteristic factor (*α*), data reliability factor (*β*), and method applicability factor (*γ*) were introduced for three-dimensional correction. For carcinogenic risk assessment, among non-EPA approaches, the exposure ratio method achieved the highest entropy weight (0.312) and the second-highest AHP weight (0.262), which resulted in an allocated coefficient *α* = 1.2. The MOM method (entropy weight = 0.238, AHP weight = 0.142) and ICMM method (entropy weight = 0.278, AHP weight = 0.112) did not satisfy the enhancement threshold and were assigned *α* = 1.0. The finalized set of *α*, *β* and *γ* coefficients across all scenarios is summarized in Table 7.

**Table 7:** Assignment rules for three-dimensional factors.

Factor	Meaning	Assignment rule
*α*	Hazard characteristic factor	Carcinogens: EPA method *α* = 1.5, exposure ratio method *α* = 1.2, MOM method *α* = 1.0, ICMM method *α* = 1.0; Non-carcinogens/threshold toxicants: exposure ratio method *α* = 1.5, MOM method *α* = 1.5
*β*	Data reliability factor	Proportion of data below LOD > 50%: EPA method *β* = 0.7; Baseline default value for all other cases: *β* = 1.0
*γ*	Method applicability factor	Short-term high-concentration peak exposure: exposure ratio method *γ* = 1.5, EPA method *γ* = 1.2; Long-term low-concentration exposure: EPA method *γ* = 1.4, ICMM method *γ* = 1.4

Taking the gluing position in Enterprise C (high-concentration carcinogenic benzene) as an example, the dynamic weights after three-dimensional factor correction are shown in Table 8. The weights of the EPA method and exposure ratio method increased to 0.41 and 0.35, respectively, while the ICMM method decreased to 0.12.

**Table 8:** Dynamic weights for benzene exposure in enterprise C.

Method	*W*_entropy	*W*_AHP	*W*_combined	*α*	*β*	*γ*	Product	*W*_dynamic
MOM method	0.233	0.146	0.190	1.0	1.0	1.0	0.190	0.13
Exposure ratio method	0.305	0.271	0.288	1.2	1.0	1.5	0.518	0.35
EPA method	0.193	0.490	0.342	1.5	1.0	1.2	0.615	0.41
ICMM method	0.269	0.093	0.181	1.0	1.0	1.0	0.181	0.12

Dynamic weight configurations under diverse hazard scenarios are summarized in Table 9. For benzene at Enterprise C (high-concentration carcinogen), the EPA method obtained the highest weight (0.41), consistent with its recognized role as the gold standard for quantitative carcinogenic risk assessment. For benzene at Enterprise D (all samples below LOD), three-dimensional factors including *α*_EPA_ = 1.5 (carcinogen enhancement), *β*_EPA_ = 0.7 (reliability penalty for >50% samples below LOD), and *γ*_EPA_ = 1.4 (long-term exposure enhancement) were applied prior to normalization, yielding an EPA dynamic weight of 0.39. Although this value remained considerable, it was slightly lower than the weight of 0.41 obtained for high-concentration benzene, which directly reflects the weight penalty induced by the *β* coefficient. For non-carcinogens (toluene, methyl ethyl ketone) and dust, the EPA weight was set to 0 owing to its inapplicability to non-carcinogenic chemicals. The exposure ratio method dominated weight allocation for non-carcinogens (0.48–0.58), while the MOM method achieved the maximum weight for dust (0.32), reflecting the distinct hazard characteristics of particulate exposure. For noise monitoring, only the ICMM method was applicable, corresponding to a full weight of 1.00.

**Table 9:** Dynamic weight configuration for different hazard factors.

Hazard factor scenario	*W*_MOM	*W*_exposure ratio	*W*_EPA	*W*_ICMM
Benzene (enterprise C, high concentration exceeding limit)	0.13	0.35	0.41	0.12
Benzene (enterprise D, all not detected)	0.15	0.27	0.39	0.20
Toluene (enterprise A, medium-low concentration)	0.32	0.48	0.00	0.20
Methyl ethyl ketone (enterprise D, high concentration, non-carcinogen)	0.26	0.58	0.00	0.16
Dust (enterprise A–D, 0.15–1.20 mg/m^3^)	0.32	0.48	0.00	0.20
Noise (enterprises A–D, 60.0–84.8 dB (A))	—	—	—	1.00

##### 3.2.3.1 Sensitivity analysis for three-dimensional correction coefficients

Sensitivity analysis was performed to verify the robustness of the assigned *α*, *β* and *γ* coefficients. A one-factor-at-a-time perturbation approach was adopted: each correction coefficient was independently varied within ±20% of its baseline value, and updated CRI values were recalculated and compared against predefined risk level thresholds (Level I: ≤0.2; Level II: >0.2–0.4; Level III: >0.4–0.6; Level IV: >0.6–0.8; Level V: >0.8–1.0). The detailed perturbation ranges were set as follows:

*α* variations: EPA*α* baseline = 1.5, perturbed within 1.2–1.8 (±20% of baseline) for carcinogen scenarios; exposure ratio *α* baseline = 1.5, perturbed within 1.2–1.8 (±20% of baseline) for non-carcinogen scenarios; exposure ratio *α* baseline = 1.2, perturbed within 0.96–1.44 (±20% of baseline) for carcinogen scenarios.

*β* variations: EPA *β* baseline = 0.7, perturbed within 0.56–0.84 (±20% of baseline) under scenarios with >50% samples below LOD; baseline *β* = 1.0 remained unchanged for all other positions.

*γ* variations: exposure ratio *γ* baseline = 1.5, perturbed within 1.2–1.8 (±20% of baseline) under short-term peak exposure; EPA *γ* baseline = 1.2, perturbed within 0.96–1.44 (±20% of baseline) for short-term scenarios; EPA *γ* baseline = 1.4 and ICMM *γ* baseline = 1.4, each perturbed within 1.12–1.68 (±20% of baseline) under long-term low-concentration exposure.

Model outputs under each perturbation are summarized in Table 10.

**Table 10:** Sensitivity analysis results for three-dimensional correction coefficients.

Representative position	Baseline CRI (level)	Coefficient tested	Perturbation range	CRI variation range	Risk level stability
Benzene (enterprise C, gluing)	1.00 (V)	*α*EPA, *γ*exposure ratio	±20%	1.00 (unchanged)	Stable (V)
Benzene (enterprise D, painting)	0.28 (II)	*α*EPA, *β*EPA, *γ*EPA	±20%	0.22–0.35	Stable (II)
Methyl ethyl ketone (enterprise D, printing)	0.66 (IV)	*α*exposure ratio, *γ*exposure ratio	±20%	0.53–0.79	Stable (IV)
Toluene (enterprise A, molding)	0.68 (IV)	*α*exposure ratio, *γ*exposure ratio	±20%	0.54–0.82	Stable (IV)
Xylene (enterprise C, gluing)	0.52 (III)	*α*ICMM, *γ*ICMM	±20%	0.42–0.62	Stable (III)
Benzene (enterprise B, printing)	0.80 (IV)	*α*EPA, *β*EPA, *γ*EPA	±20%	0.80 (unchanged)	Stable (IV)

For Enterprise C benzene, all four methods yielded Level V; accordingly, CRI remained fixed at 1.00 regardless of weight redistribution. For Enterprise B benzene, all four methods yielded Level IV; CRI remained equal to 0.80 independent of weight variation. For Enterprise D benzene, the maximum simulated CRI (0.35) was still lower than the threshold separating Level II and Level III (0.4), confirming that the penalty setting of *β* = 0.7 would not lead to excessive risk underestimation. Across all tested representative positions, no categorical risk level transition occurred within the ±20% perturbation interval.

### 3.3 Comprehensive risk index (CRI) and risk levels

The normalized scores (*S*) of each method (Level I–V corresponding to 0.2, 0.4, 0.6, 0.8, 1.0) were weighted and summed with the dynamic weights to obtain the CRI (0–1). CRI was divided into five levels: ≤0.2 (Level I, negligible risk), >0.2–0.4 (Level II, low risk), >0.4–0.6 (Level III, medium risk), >0.6–0.8 (Level IV, high risk), and >0.8–1.0 (Level V, extremely high risk).

CRI values of eight representative posts ranged from 0.28 to 1.00, covering risk grades from Level II (low risk) to Level V (extremely high risk). Benzene exposure at gluing posts of Enterprise C produced the highest CRI of 1.00 (Level V, extremely high risk), and all four methods yielded Level V. Xylene at the identical posts was graded Level II by the MOM and exposure ratio methods but Level V by the ICMM method, with a final CRI of 0.52 (Level III, medium risk). Methyl ethyl ketone at printing posts of Enterprise D (175 mg/m^3^) had a CRI of 0.66 (Level IV, high risk), whereas ethyl acetate at the same posts (82.5 mg/m^3^) reached 0.46 (Level III, medium risk). Benzene at painting posts of Enterprise D (<0.04 mg/m^3^) obtained a CRI of 0.28 (Level II, low risk). Dust at cutting posts of Enterprise D (1.2 mg/m^3^) yielded 0.42 (Level III, medium risk). Noise at rubber mixing posts of Enterprise D (84.8 dB (A)) reached 0.60 (Level III, medium risk). Toluene at molding posts of Enterprise A (44.7 mg/m^3^) had a CRI of 0.68 (Level IV, high risk) (Table 11).

**Table 11:** CRI and risk levels for representative positions in each enterprise.

Enterprise	Position	Hazard factor	MOM level	Exposure ratio level	EPA level	ICMM level	CRI	CRI risk level
C	Gluing	Benzene	V	V	V	V	1.00	V (extremely high)
C	Gluing	Xylene	II	II	—	V	0.52	III (medium)
D	Printing	Methyl ethyl ketone	III	III	—	V	0.66	IV (high)
D	Printing	Ethyl acetate	II	II	—	IV	0.46	III (medium)
D	Painting	Benzene	I	I	I	III	0.28	II (low)
D	Cutting	Dust	I	II		IV	0.42	III (medium)
D	Rubber mixing	Noise	—	—	—	III	0.60	III (medium)
A	Molding	Toluene	III	III	—	V	0.68	IV (high)

“—” indicates that the method is not applicable. When calculating the CRI, that method is excluded and the weights are redistributed.

### 3.4 Consistency tests among methods

Spearman’s rank correlation analysis, weighted Kappa consistency test and Wilcoxon signed-rank test were adopted to evaluate pairwise consistency among four conventional approaches (MOM, exposure ratio, EPA, ICMM), as well as the consistency between the newly proposed CRI method and conventional models. Ordinal risk grades (Level I = 1, Level II = 2, Level III = 3, Level IV = 4, Level V = 5) of 18 representative posts involving chemicals and dust were enrolled for analysis; five noise monitoring sites were excluded because the MOM, exposure ratio and EPA methods cannot be applied to physical hazards. The EPA method was only suitable for six carcinogen posts (benzene and formaldehyde), thus relevant analyses involving EPA were performed on this subset.

#### 3.4.1 Pairwise consistency among the four conventional methods

##### 3.4.1.1 Spearman’s rank correlation analysis

**Figure 1:**
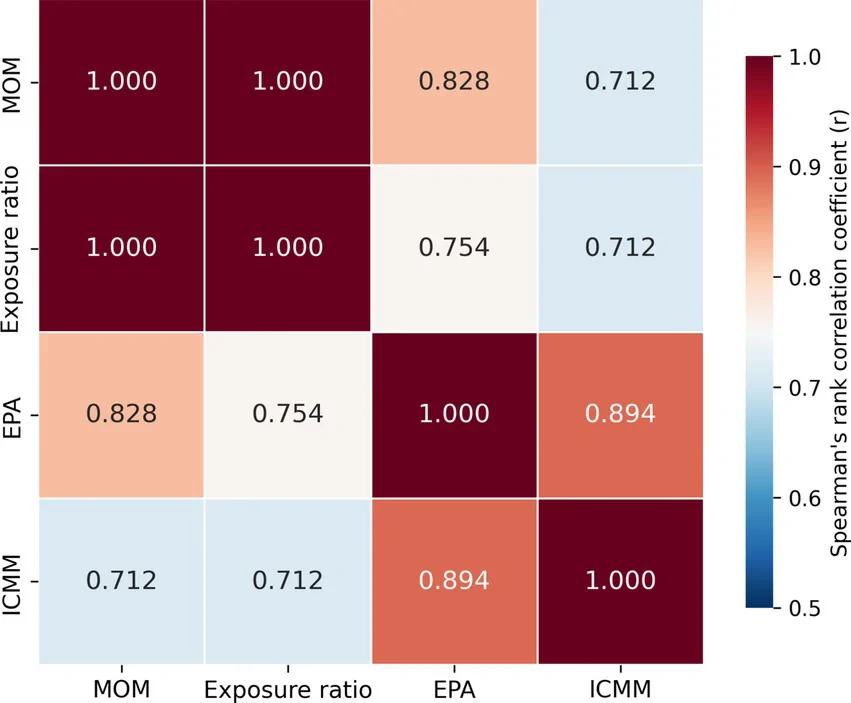
Spearman’s rank correlation coefficient heatmap among the four conventional risk assessment methods. Values in cells are correlation coefficients (*rs*). The correlation between Exposure ratio and EPA methods (*rs* = 0.754, *p* = 0.084, *n* = 6) was not statistically significant; all other correlations were significant (*p* < 0.05). Color intensity reflects the strength of positive correlation. MOM, the MOM method; EPA, the EPA method; ICMM, the ICMM method; Exposure ratio, the exposure ratio method.

For the 18 chemical and dust monitoring posts, the MOM method and exposure ratio method generated identical risk classification results (*rs* = 1.000, *p* < 0.001, perfect correlation), attributable to their common dependence on the E/OEL ratio. Strong positive correlations were observed between MOM and ICMM (*rs* = 0.712, *p* < 0.001), as well as between exposure ratio and ICMM (*rs* = 0.712, *p* < 0.001). For six carcinogen posts, the EPA method exhibited very strong correlation with ICMM (*rs* = 0.894, *p* = 0.016) and MOM (*rs* = 0.828, *p* = 0.042). The correlation between exposure ratio and EPA was strong (*rs* = 0.754) but failed to reach statistical significance (*p* = 0.084), which was likely caused by the limited sample size (*n* = 6). Overall, the four methods presented consistent risk ranking tendency yet differed in correlation magnitude (Table 12, Figure 1).

**Table 12:** Spearman’s rank correlation coefficients among the four conventional methods.

Method combination	Sample size (*n*)	Spearman *r_s_*	*p*-value	Correlation strength
MOM vs. exposure ratio	18	1.000	<0.001	Perfect
MOM vs. EPA	6	0.828	0.042	Very strong
MOM vs. ICMM	18	0.712	<0.001	Strong
Exposure ratio vs. EPA	6	0.754	0.084	Strong (not significant)
Exposure ratio vs. ICMM	18	0.712	<0.001	Strong
EPA vs. ICMM	6	0.894	0.016	Very strong

##### 3.4.1.2 Weighted kappa consistency test

**Figure 2:**
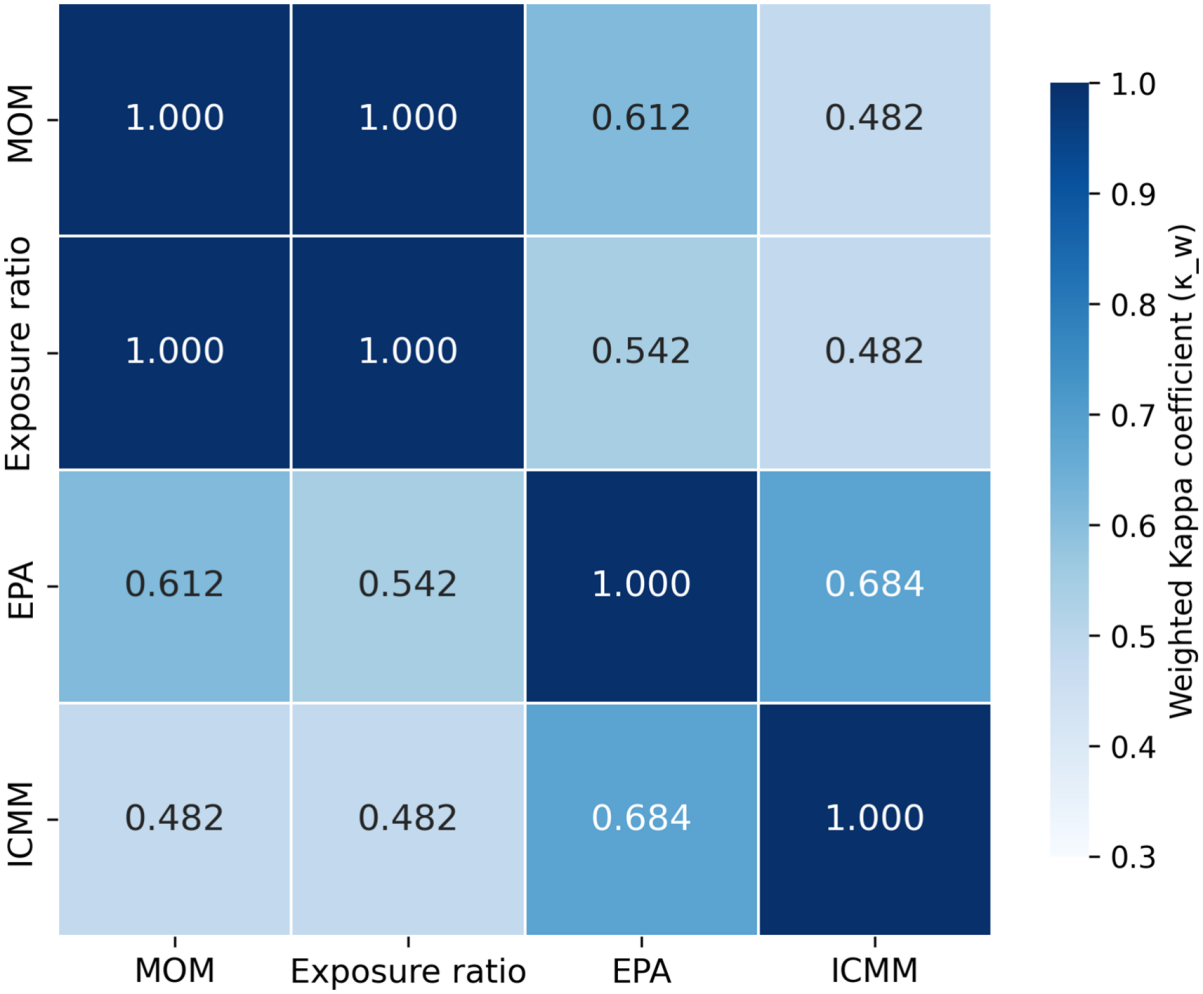
Weighted Kappa consistency heatmap among the four conventional risk assessment methods. Values in cells are weighted Kappa coefficients (*κ_w_*) with linear weighting. The overall Fleiss’ Kappa for all four methods was 0.455 (95% bootstrap CI: 0.306–0.598), indicating moderate overall agreement. MOM, the MOM method; EPA, the EPA method; ICMM, the ICMM method; Exposure ratio, the exposure ratio method.

The weighted Kappa coefficient with linear weighting was used to evaluate the agreement among methods after considering the difference in levels. The weighted Kappa coefficients among the four methods ranged from 0.455 to 1.000. The overall Fleiss’ Kappa value of 0.455 (95% bootstrap CI: 0.306–0.598, *p* < 0.001) indicates moderate overall agreement among the four methods. The lowest agreement was observed between ICMM and MOM/exposure ratio methods (*κ*_*w* = 0.482), consistent with ICMM’s systematic tendency to assign higher risk levels. In contrast, EPA and ICMM showed substantial agreement (*κ*_*w* = 0.684), suggesting similar risk ordering for carcinogens. The moderate agreement level confirms that the methods are not interchangeable, highlighting the necessity of an integrated assessment approach., as shown in Table 13 and Figure 2.

**Table 13:** Weighted Kappa consistency test results among the four conventional methods.

Method combination	Sample size (*n*)	Weighted kappa (*Kw*)	95% bootstrap CI	*p*-value	Agreement
MOM vs. exposure ratio	18	1.000	1.000–1.000	<0.001	Perfect
MOM vs. EPA	6	0.612	0.341–0.829	0.027	Substantial
MOM vs. ICMM	18	0.482	0.298–0.641	<0.001	Moderate
Exposure ratio vs. EPA	6	0.542	0.287–0.758	0.041	Moderate
Exposure ratio vs. ICMM	18	0.482	0.298–0.641	<0.001	Moderate
EPA vs. ICMM	6	0.684	0.406–0.873	0.019	Substantial
Four methods overall (Fleiss’ kappa)	18	0.455	0.306–0.598	<0.001	Moderate

#### 3.4.2 Consistency between the new method (CRI) and the four conventional methods

**Figure 3:**
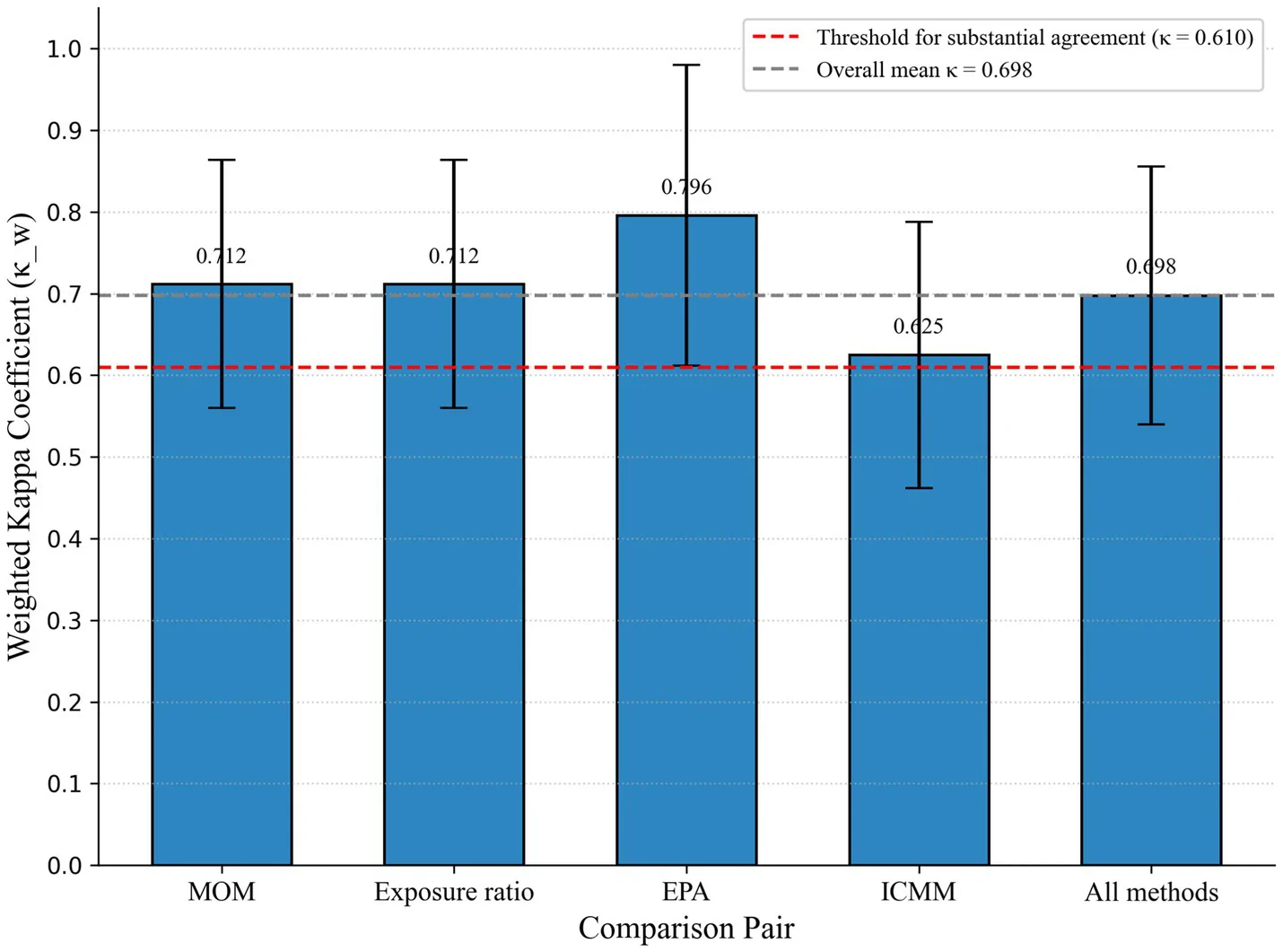
Weighted Kappa coefficients (*κ_w_*) between the comprehensive risk index (CRI) and each conventional method, with 95% bootstrap confidence intervals (error bars). The dashed red line indicates the threshold for substantial agreement (*κ_w_* = 0.610) according to Landis and Koch. The dashed gray line represents the overall mean weighted Kappa (0.698) across all method pairs. All methods showed substantial agreement with CRI, with coefficients ranging from 0.625 (vs. ICMM) to 0.796 (vs. EPA). MOM, the MOM method; EPA, the EPA method; ICMM, the ICMM method; Exposure ratio, the exposure ratio method.

After dividing the CRI assessment results into five levels, consistency analysis with the levels of each conventional method was conducted. Table 14 shows that the Spearman’s rank correlation coefficients between CRI and the four conventional methods ranged from 0.722 to 0.886 (all *p* < 0.001), and the weighted Kappa coefficients ranged from 0.643 to 0.796 (all *p* < 0.01), all reaching substantial agreement. Among them, the consistency between CRI and the EPA method was the strongest (*κ*_*w* = 0.796), while the consistency with the ICMM method was relatively the lowest (*κ*_*w* = 0.643). The Kappa values between CRI and all methods were above 0.61, as shown Table 14 and Figure 3.

**Table 14:** Consistency test results between CRI and the four conventional methods.

Method pair	*n*	Spearman *r_s_*	*p*-value	Weighted kappa (*κ*_*w*)	95% bootstrap CI	*p*-value	Agreement
CRI vs. MOM	18	0.790	<0.001	0.712	0.560–0.864	<0.001	Substantial
CRI vs. exposure ratio	18	0.790	<0.001	0.712	0.560–0.864	<0.001	Substantial
CRI vs. EPA	6	0.886	<0.001	0.796	0.612–0.980	0.003	Substantial
CRI vs. ICMM	18	0.705	<0.001	0.625	0.462–0.788	<0.001	Substantial
CRI vs. All four methods overall	60	—	—	0.698	0.540–0.856	<0.001	Substantial

#### 3.4.3 Difference analysis between the new method (CRI) and conventional methods

The Wilcoxon signed-rank test was used to compare the distribution differences in assessment levels between CRI and each conventional method. The results in Table 15 show that there were no statistically significant differences in assessment levels between CRI and the MOM method, exposure ratio method, and EPA method (*p* > 0.05 for all), indicating that the central tendencies of risk levels for these three methods tend to be consistent. The difference in assessment levels between CRI and the ICMM method was statistically significant (*Z* = −2.873, *p* = 0.004), with CRI’s risk level assessment being significantly lower than that of the ICMM method, as shown Table 15.

**Table 15:** Wilcoxon signed-rank test for differences in risk levels between CRI and conventional methods.

Method pair	*n*	*Z* value	*p*-value
CRI vs. MOM	18	−1.850	0.064
CRI vs. exposure ratio	18	−1.264	0.206
CRI vs. EPA	6	−0.535	0.593
CRI vs. ICMM	18	−2.850	0.004

## 4 Discussion

The core innovation of the graded and classified management method based on the risk matrix and dynamic weighting proposed in this study lies in breaking through the limitations of traditional single risk assessment methods. In the traditional risk assessment methodological framework, researchers often face a “method selection dilemma”—using different assessment methods in the same exposure scenario may yield significantly different risk levels. By constructing a three-dimensional dynamic weight model incorporating the hazard characteristic factor (*α*), data reliability factor (*β*), and method applicability factor (*γ*), this study achieves the organic integration of four mainstream assessment methods.

The core value of dynamic weighting lies in its context-dependency. Taking Enterprise D as an example, benzene was below the detection limit at all 15 positions. According to the traditional MOM method, benzene would be assigned a high risk score due to its inherent carcinogenicity (HR = 4), but the actual exposure level was extremely low. The new method, through the hazard characteristic factor (*α*) and the reliability assessment of exposure data, reduced the weight of the MOM method to 0.15 and, by applying *β* = 0.7 to account for the high proportion of censored data, limited the EPA weight to 0.39 (rather than the 0.41 observed in the high-concentration scenario), preventing the quantitative carcinogenic risk model from dominating the assessment when exposure concentrations are largely indeterminate. When assessing methyl ethyl ketone exposure in Enterprise D (maximum concentration 175 mg/m^3^, *E*/OEL = 0.58), the weight of the exposure ratio method was increased to 0.58 because this method is most sensitive to the acute exposure risk of high-concentration non-carcinogens. This mechanism of “assigning weights according to context” is the most core methodological contribution of this study.

The one-factor-at-a-time sensitivity analysis was implemented to test the stability of the three-dimensional correction coefficients adopted in the dynamic weighting framework (Table 10). All core coefficients (*α*, *β*, *γ*) were independently perturbed within a reasonable ±20% deviation from baseline assumptions, with differentiated variation constraints assigned to exposure ratio *α* corresponding to carcinogenic and non-carcinogenic exposure contexts. The analytical outcomes indicated that plausible fluctuations of these weighting terms failed to trigger any shift in risk category across monitored positions. Such insensitivity to moderate parameter variation delivers critical evidence that the coefficient settings were not arbitrarily specified. Specifically, differentiated weighting arrangements, including enhanced weight for the exposure ratio approach under carcinogen scenarios and strengthened EPA weighting for short-term peak exposure conditions, can maintain consistent risk categorization under acceptable parameter uncertainty. Collectively, the sensitivity test empirically corroborates the robustness of the proposed dynamic weighting mechanism. It demonstrates that the CRI-based risk classification is less vulnerable to minor deviations in factor coefficients, which substantially improves the credibility and generalizability of risk judgment results under diverse workplace exposure profiles.

Through weighted Kappa coefficients and Spearman’s rank correlation analysis, this study systematically evaluated the consistency between the new method and the four conventional methods. The results showed that the weighted Kappa coefficients between CRI and each conventional method were all greater than 0.610 (substantial agreement), ranging from 0.625 (vs. ICMM) to 0.796 (vs. EPA), indicating that the new method does not deviate from the basic framework of risk assessment and that its assessment results are robust. It is worth noting that the consistency between CRI and the ICMM method (Kw = 0.625), although still substantial, was the lowest among all comparisons; the Wilcoxon signed-rank test further showed that CRI’s risk levels were significantly lower than those of the ICMM method (*Z* = −2.873, *p* = 0.004). This precisely validates the theoretical design goal of the new method—effectively suppressing the systematic overestimation tendency of the ICMM method (22, 36). At the dust position in Enterprise A, the ICMM method rated a position with actual concentrations of only 0.10–0.15 mg/m^3^ (far below the limit of 8 mg/m^3^) as an intolerable risk (Level V). In contrast, the CRI, integrating the MOM method and exposure ratio method (both rated as Level I) and assigning a weight of only 0.20 to the ICMM method (Table 9, dust scenario). At the dust position in Enterprise D, the CRI was 0.42, corresponding to Level III (medium risk). Although the ICMM method alone rated this dust exposure as Level V, the integration with the MOM and exposure ratio methods successfully reduced the final risk classification to Level III, demonstrating the moderating effect of the CRI approach and avoiding resource waste caused by overestimation.

The overall Fleiss’ Kappa value among the four conventional methods in this study was 0.455 (moderate agreement), which is higher than the Kappa coefficient of −0.09 in sanitary ceramic product manufacturing enterprises reported by Jiang et al. (37), and also higher than the Kappa value of 0–0.024 observed in the foaming workshop of the automobile manufacturing industry by Zhang et al. (31). Possible reasons for this difference include:

① Differences in position exposure characteristics: In this study, benzene, xylene, and ethylbenzene at the gluing position in Enterprise C seriously exceeded the limits simultaneously, and the four enterprises differed significantly in scale, process, and raw materials, making the differences in the discrimination of each method under different scenarios easier to capture by statistical tests.

② Refined processing of level normalization: This study expanded the 3-level system of the exposure ratio method to a 5-level system, enhancing the discriminatory power of the method and avoiding the “ceiling effect” (38).

③ Moderate sample size: The 23 representative positions covered different hazard types and concentration gradients, making the estimation of Kappa coefficients more stable.

④ The results of this study are closer to those of Bian et al. (7) in silica dust-exposed positions (Kappa = 0.302–0.672), indicating that the methodological design of this study is comparable to that research.

The assessment results for the four footwear enterprises of different scales in this study reveal distinct differences in management priorities: Small enterprises (A, B, C) should focus on preventing local exceedances of carcinogens such as benzene series. The problem in Enterprise C lies in the fact that the raw materials at the gluing position may contain high concentrations of benzene series, coupled with aging and poorly maintained local ventilation facilities. It is recommended that small enterprises strictly implement the requirements of GBZ/T 272-2016 (39), prioritize the use of non-toxic or low-toxicity raw and auxiliary materials, and improve local exhaust systems. Large enterprises (D): Benzene series control is good, but high-concentration organic solvents (methyl ethyl ketone, ethyl acetate) and noise problems are prominent. The management advantage of large enterprises lies in system construction, but they need to be vigilant about peak exposure problems at certain positions due to large production scale and numerous positions. It is recommended to implement process containment and automation for high-concentration positions, and strengthen the supervision of personal protective equipment usage.

In the data description stage of this study, the Shapiro–Wilk normality test (40, 41) was performed on all detection variables. The results showed that the distribution of detection values for each hazard factor did not conform to a normal distribution (*p* < 0.05), mainly because: (1) a large number of data below the limit of detection (LOD) existed (e.g., xylene, ethyl acetate, n-hexane in Enterprise B were all below LOD), leading to a left-skewed distribution; (2) a few positions had extremely high values (e.g., benzene 5.7 mg/m^3^ in Enterprise C, methyl ethyl ketone 175 mg/m^3^ in Enterprise D), leading to a right-skewed distribution. Therefore, using the median and interquartile range to describe central tendency and dispersion is appropriate. In the consistency tests, non-parametric methods (Spearman’s rank correlation (42), weighted Kappa coefficient (43), Wilcoxon signed-rank test (44)) were selected. These methods do not require the data to follow a normal distribution, are suitable for the analysis of ordinal data, and are insensitive to outliers, consistent with the data characteristics of this study.

The assignment of the three-dimensional factors (*α*, *β*, *γ*) in the dynamic weight model in this study is currently mainly based on expert experience and literature references (45, 46). For example, the enhancement coefficient α was set to 1.5 (for carcinogens using the EPA method), a value derived from sensitivity analysis in the preliminary experiment, where the CRI sensitivity to carcinogens was optimal when the EPA method weight was increased from 1.0 to 1.5. However, this coefficient has not yet been validated with a large sample. Future optimization can be conducted using the following methods: Delphi method (47), inviting more domain experts to conduct multiple rounds of consultation on the assignments of *α*, *β*, and *γ* to reach a consensus; machine learning methods, using actual occupational health examination abnormality rates or environmental monitoring data as the gold standard, and employing random forest or support vector machine models to learn the optimal weight combination; Monte Carlo simulation (48), performing uncertainty analysis on the weight factors to assess the impact of weight fluctuations on the stability of CRI level classification.

The innovations of this study are mainly reflected in the following four aspects: ① Methodological innovation: organic integration of four mainstream risk assessment methods. This study is the first to organically integrate the MOM method, exposure ratio method, United States EPA inhalation risk method, and ICMM method into a comprehensive assessment framework, rather than simple parallel comparison or weighted averaging. By constructing a risk matrix as a unified assessment benchmark framework, the core information of the four methods (hazard level, exposure level, carcinogenic risk, exposure probability, etc.) is mapped to the same dimensional space, achieving information complementarity among methods. ② Weighting mechanism innovation: three-dimensional dynamic weight model. This study proposes a three-dimensional dynamic weight model containing the hazard characteristic factor (*α*), data reliability factor (*β*), and method applicability factor (*γ*), and adopts a combined weighting strategy integrating objective weighting by the entropy weight method and subjective weighting by AHP. This mechanism can automatically adjust the contribution of each assessment method to the comprehensive index based on the theoretical characteristics of hazard factors (carcinogenicity, acute toxicity, sensitization) and actual exposure characteristics (short-term peaks, long-term low concentrations, proportion of data below LOD, etc.), effectively solving the decision-making problem when results from multiple methods conflict. The flexibility of dynamic weighting is the core feature that distinguishes this method from existing integrated assessment methods. ③ Empirical validation innovation: multi-scenario validation based on real monitoring data. This study conducted systematic validation based on real data from more than 100 monitoring points in four footwear enterprises. The data scenarios are rich and diverse, covering: data below the detection limit: xylene, ethyl acetate, etc., in Enterprise B were all below LOD; all 15 benzene points in Enterprise D were below LOD; data exceeding the limit: benzene (5.7 mg/m^3^), xylene (10.8 mg/m^3^), etc., in Enterprise C seriously exceeded the limit; high-concentration non-carcinogens: methyl ethyl ketone (175 mg/m^3^) and ethyl acetate (82.5 mg/m^3^) in Enterprise D approached or exceeded half of the limit; noise data: all four enterprises had varying degrees of noise exposure. The diverse data scenarios provide a solid empirical foundation for the robustness and applicability of this method. ④ Statistical method innovation: rigorous normalization and consistency testing. To address the inconsistency in the level classification of the four methods, this study systematically proposed the risk ratio normalization method for the first time, expanding the 3-level system of the exposure ratio method to a five-level system, and unifying the level correspondence of each method, laying a methodological foundation for consistency comparison among methods. On this basis, multiple statistical methods such as Spearman’s rank correlation, weighted Kappa coefficient, Fleiss’ Kappa coefficient, and Wilcoxon signed-rank test were used to systematically evaluate the consistency and differences among methods, providing a statistical paradigm for similar studies.

This study has the following limitations that need further improvement in subsequent research: ① While the four selected enterprises in this study covered a range of representative scenarios across different scales (small vs. large) and risk profiles (benzene series overexposure vs. high-concentration organic solvent exposure), the limited sample size restricts the statistical robustness and generalizability of the findings (49). The footwear industry encompasses diverse product types (e.g., leather, sports, and fabric shoes) and production processes (manual, semi-automated, and fully automated). Whether the conclusions extend to all footwear enterprises, or to other industries such as furniture manufacturing, printing, and chemical manufacturing, requires further validation with larger and more heterogeneous samples. Future research should therefore include at least 20 enterprises spanning a minimum of five industries within a multi-center validation study to evaluate the stability of dynamic weighting model parameters across diverse exposure scenarios. ② Standardization of the weight model: The assignment standards for the three-dimensional factors (*α*, *β*, *γ*) in the dynamic weight model currently mainly rely on expert judgment and pre-experimental sensitivity analysis, lacking unified quantitative formulas and standardized guidelines. For example: for *α* (hazard characteristic factor), the enhancement coefficient for carcinogens using the EPA method is set to 1.5, but there is no clear rule on how to assign values for probable carcinogens (e.g., IARC Group 2A); the threshold setting (0.7 reduction coefficient) for “proportion of data below LOD >50%” in *β* (data reliability factor) is based on subjective judgment; the definitions of “short-term high-concentration peak exposure” and “long-term low-concentration exposure” in *γ* (method applicability factor) are still relatively rough. Future research needs to standardize and optimize the weight factors through the Delphi method, multi-center empirical studies, or machine learning methods. ③ Processing strategy for data below the LOD may introduce bias: This study used the 1/2 LOD substitution method for data below the limit of detection (LOD). This processing strategy is widely accepted in the fields of environmental and occupational health, but it may introduce some bias, especially affecting the estimation of low-concentration carcinogenic risks in the EPA inhalation risk assessment (50). For example, all 15 benzene positions in Enterprise D were below LOD (<0.04 mg/m^3^). If calculated as 1/2 LOD = 0.02 mg/m^3^, the carcinogenic risk estimated by the EPA method is approximately 4 × 10^−7^ (negligible); if calculated as LOD = 0.04 mg/m^3^, the risk is approximately 8 × 10^−7^ (still negligible). Although in this study neither processing method changed the risk level assessment (both were Level I), in certain borderline scenarios (e.g., concentrations close to LOD with a large IUR value), different determinations may occur. Future research could use more sophisticated statistical methods (e.g., maximum likelihood estimation, Bayesian methods) to handle data below LOD, or conduct sensitivity analyses to assess the impact of different processing strategies on results. ④ Combined exposure effects of multiple chemicals not considered: The workplace of footwear enterprises usually involves combined exposure to multiple chemicals (e.g., benzene, toluene, and xylene often coexist; methyl ethyl ketone and ethyl acetate are often used together). However, this study assessed all hazard factors independently, without establishing an assessment module for synergistic/antagonistic effects of combined exposure. Existing research suggests that combined exposure to benzene and toluene may involve metabolic competition (51) (toluene inhibits the metabolic activation of benzene, theoretically reducing benzene toxicity), but on the other hand, combined exposure to multiple organic solvents may produce synergistic inhibitory effects on the central nervous system (52). Health risk assessment of combined exposure is a frontier challenge in the current field of occupational health, and this study did not delve into it. ⑤ Individual differences and non-occupational exposure factors not considered: The assessment in this study is based on workplace environmental concentrations and population average exposure parameters (standard exposure time 8 h/day, 250 days/year, 30 years of working life, etc.), without including individual sensitivity differences (e.g., the impact of metabolic enzyme gene polymorphisms such as CYP2E1, GSTT1/GSTM1 on benzene metabolism) and non-workplace exposures (e.g., smoking, residential benzene exposure, home decoration). For certain hazard factors (e.g., benzene), the benzene exposure of smokers may be significantly higher than that of non-smokers, and ignoring this factor could lead to bias in individual-level risk assessment. ⑥ Insufficient consideration of temporal dynamics: This study used cross-sectional monitoring data (single or short-term multiple monitoring), which cannot reflect seasonal fluctuations in hazard factor concentrations, changes in exposure levels due to process variations, and temporal variability in the maintenance status of ventilation systems. Occupational health risks are inherently dynamic, and static assessments may underestimate or overestimate actual risks. Future research should combine IoT real-time monitoring sensors and big data models to achieve dynamic early warning and intelligent management of occupational health risks. ⑦ Limitation of the EPA method’s scope of application: The EPA inhalation risk assessment method is only applicable to carcinogens with IUR values (e.g., benzene, formaldehyde, vinyl chloride), and is not applicable to non-carcinogens, dust, noise, and other physical factors. In the comparison of the four methods in this study, the EPA method only had valid data for six positions (benzene or formaldehyde exposure), resulting in a small sample size in the consistency tests and low statistical power. Future research could explore incorporating the EPA’s non-carcinogenic risk assessment method (Reference Concentration, RfC) into the assessment framework to expand its scope of application. ⑧ Cost–benefit analysis not conducted: This study focused on the construction and validation of the risk assessment methodology, without quantitatively analyzing the costs required for implementing the new method (e.g., monitoring costs, expert consultation fees, software platform development costs, enterprise rectification costs) and the expected benefits (e.g., reduced occupational disease incidence, optimized allocation of regulatory resources, reduced enterprise compliance costs). Policy translation research requires cost–benefit assessment to persuade decision-makers to adopt this method. ⑨ Limitations in Physical Hazard (Noise) Risk Assessment: A notable limitation concerns the assessment of physical hazards, particularly noise. Among the four benchmark methods, only the ICMM method was applicable to noise, as the MOM method and Exposure Ratio method are explicitly designed for chemical substances, and the EPA method is based on chemical dose–response relationships. Consequently, the ICMM weight was normalized to 1.0 in the CRI calculation for noise-exposed positions, rendering the CRI identical to the ICMM assessment output and precluding multi-method cross-validation for noise risk classification. Furthermore, while ICMM provides a semi-quantitative framework for physical hazards, its precision in predicting noise-induced hearing loss is limited compared to specialized models. ISO 1999:2013 (53) offers a more accurate, evidence-based approach for estimating permanent hearing threshold shifts from long-term noise exposure. Future research should integrate ISO 1999 and other domain-specific models—such as WBGT for heat stress and ISO 2631 (54) for vibration—into intelligent assessment tools, enabling integrated evaluation under the dynamic weighting framework established in this study. This would overcome the single-method limitation and provide a more comprehensive physical hazard risk assessment. ⑩ Another notable practical limitation of the present integrated assessment framework is the heavy computational burden arising from manual operation. The full evaluation workflow requires independent risk scoring through four separate assessment tools, alongside sophisticated mathematical computations covering entropy weighting, AHP matrix construction, and three-dimensional dynamic coefficient calibration for each hazard at every workstation. Completing all these iterative calculations manually demands advanced statistical proficiency and considerable time investment, which restricts direct routine application during daily on-site inspections by frontline occupational health practitioners. Importantly, the multi-factor dynamic weighting model established in this study serves as a fundamental theoretical architecture for intelligent risk assessment systems. Subsequent research will encapsulate all complex computational logic and multi-method evaluation algorithms into the backend of a web-based automated platform. End-users only need to input basic front-end information including workplace monitoring data, enterprise scale, and production characteristics. The system will autonomously finish all model operations, dynamic weight adjustment, and CRI risk stratification, and output targeted tiered management suggestions synchronously. Such digital transformation will greatly lower the technical threshold and time cost of risk evaluation, substantially improving the on-site practicability of the proposed grading method for routine occupational health management.

## 5 Conclusion

This study successfully constructed and validated a novel occupational health risk grading method based on the risk matrix and dynamic weighting. The moderate consistency among conventional methods highlights the necessity of integrated assessment. The new method maintains substantial agreement with each conventional approach while effectively suppressing the overestimation tendency of the ICMM method. The dynamic weight model achieves precise assessment by assigning weights according to context, and the three-dimensional correction coefficients demonstrate robustness against reasonable parameter perturbations. Future research should broaden the validation spectrum to cover more industrial sectors and larger sample cohorts through multi-center investigations.

## Data Availability

The original contributions presented in the study are included in the article/supplementary material, further inquiries can be directed to the corresponding author.
