# Correction: Construction and application of an occupational health risk grading method based on risk matrix and dynamic weighting: a case study of footwear manufacturing enterprises

**DOI:** 10.3389/fpubh.2026.1999091

**Published:** 2026-09-24

**Authors:** Hongying Bian, Xin Wang, Yiwen Dong, Ning Kang, Lin Zhang

**Affiliations:** National Institute of Occupational Health and Poison Control, Chinese Center for Disease Control and Prevention & Chinese Academy of Preventive Medicine, Beijing, China

**Keywords:** dynamic weighting, footwear manufacturing enterprise, occupational health, risk assessment, risk matrix

The funder “The Investigation of Special Occupational Environments, 012100409014260010301” to Hongying Bian was erroneously included.

The original version of this article has been updated.

